# Synthesis of Zinc Nanoparticles by the Gas Condensation Method in a Non-Contact Crucible and Their Physical–Chemical Characterization

**DOI:** 10.3390/nano14020163

**Published:** 2024-01-11

**Authors:** Artyom N. Markov, Alexander A. Kapinos, Anton N. Petukhov, Egor S. Dokin, Artem V. Emelyanov, Nataliia V. Abarbanel, Dmitriy M. Zarubin, Anna A. Golovacheva, Sergey S. Suvorov, Alexandra V. Barysheva, Pavel P. Grachev, Ilya V. Vorotyntsev, Andrey V. Vorotynstev

**Affiliations:** 1 Chemical Engineering Laboratory, Lobachevsky State University of Nizhny Novgorod, Nizhny Novgorod 603022, Russiaan.vorotyntsev@gmail.com (A.V.V.); 2 Laboratory of Smart Materials and Technologies, Mendeleev University of Chemical Technology of Russia, Miusskaya Sq. 9, Moscow 125047, Russia

**Keywords:** zinc, nanoparticles, induction flow levitation, high-temperature synthesis

## Abstract

This work explored the zinc nanoparticles obtained by the one-stage induction flow levitation method. A 10 kW tube generator with an operating frequency of 440 kHz was used. The process used 8 mm diameter zinc granules (2 g weight) with a purity of 99.9%. Zinc wire was fed to replace the evaporated metal from the granule surface. This method productivity was 30 g/h of nanoparticles. In addition, various methods were used to characterize the resulting nanoparticles: scanning electron microscopy (SEM), transmission electron microscopy (TEM), X-Ray fluorescence analysis (XRF), dynamic light scattering (DLS), porosimetry and inductively coupled plasma atomic emission spectroscopy (ICP-MS). The resulting nanoparticle size, determined by SEM and porosimetry, was 350 nm, while the size of the primary crystallites was 21 nm. The amount of impurities in the resulting nanoparticles did not exceed 1000 ppm.

## 1. Introduction

Over the past decade, the field of nanotechnology has developed rapidly [1], and products containing nanoparticles have found applications in various fields such as food processing, electronics, chemicals, cosmetics and pharmaceuticals [2]. Nanoparticles (NPs) have a size from 1 to 100 nm [3]. Their properties depend on their size and functional surface, and it is these parameters that determine their widespread use.

Nanoparticles, due to their unique properties, are widely used in the chemical industry [4], improving production processes, creating new materials [5] and improving catalysts [6]. Implementation of nanoparticles in polymers, for example, can improve the mechanical, electrical or thermal material properties, opening up new possibilities for the development of stronger, lighter and more functional materials. Nanoparticles play a key role in catalysis because of their high activity and increased surface area [7], making them effective chemical reactions catalysts. Nanoparticles, especially metals or their compounds, are used as catalysts to speed up reaction rates, which also reduces the process temperature or pressure, making it more efficient and economic [8]. They are used in filtration, purification and raw material processing, contributing to the cleaner and higher quality final product production [9].

Some of the most abundant metal nanoparticles used for catalysis include platinum, due to its stability, used in various chemical processes such as the production and oxidation of ammonia [10]. Palladium nanoparticles are often used in heterogeneous catalysts for hydrogenation reactions and organic synthesis reactions, because of their high selectivity and activity [11]. Gold nanoparticles are used for alcoholates oxidation in organic synthesis, hydrogenation and also as an electrocatalyst for electrochemical reactions [12].

All the above nanoparticles are precious metals and have a high cost. This may limit chemical process scaling using these catalyst types because of high economic costs. The transition from expensive catalysts to more accessible and cheaper alternatives, such as zinc, is a current trend in the catalysis field [13].

Zinc nanoparticles can be used as a catalyst in processes such as the synthesis, hydrogenation and oxidation of alkoxides and amino alcohols. Also, they can be used in carbon nanotube synthesis processes, which is important for electronics, materials science and the development of other technological areas [14].

Using more accessible and inexpensive metals like zinc as catalysts represents a significant step towards reducing production costs. Zinc, as a relatively available and widespread metal, can reduce limited resource dependence. The transition to zinc usage as a catalyst not only contributes to a more efficient production organization but also meets the challenges of sustainable development, minimizing the environment’s negative impact [15].

There are several methods for obtaining zinc nanoparticles that can be used in laboratory and industry. Among them: zinc grinding in ball mills, based on the zinc material’s mechanical action, which leads to nanoparticle formation because of the high energy introduced into the grinding process [16,17]; hydrothermal methods [18], including the high pressure and temperature used for zinc nanoparticle synthesis; laser ablation, based on material irradiation with laser radiation [19]; electrolysis, a process in which zinc nanoparticles are deposited on an electrode under the influence of an electric current [20]; chemical deposition [21], which involves chemical reactions leading to the formation of zinc nanoparticle thin films [22]. Each of these methods has its own unique advantages and can be selected depending on the specific requirements for particle size, structural features and intended industrial application.

However, these methods for obtaining zinc nanoparticles have limitations. Mechanical impact on the material requires a significant amount of energy, which can make this process uneconomical. In addition, product contamination may result from ball mill depreciation or the usage of additives. Hydrothermal methods, which require high temperatures and pressures, may face difficulties in process scaling. Managing high temperatures and pressures may need more resources, making commercialization difficult for this method. Laser ablation, despite providing the resulting nanoparticles at high purity, requires specialized equipment at significant costs. Regulating the ablation process may also be difficult, limiting its widespread industrial use. Electrochemical methods require complex equipment and are prone to instability. Optimal control of electrochemical processes can be challenging, and instability can affect the resulting nanoparticle quality and uniformity. Chemical deposition faces challenges in controlling particle size, which can affect the functional properties. In addition, chemical precipitation can generate toxic waste, which poses a serious problem in terms of the environmental sustainability of the production processes [23]. Producing nanoparticles using the induction flow levitation method makes it possible to heat samples in an inert atmosphere to 2500 °C and literally evaporate the metal, followed by condensation into nanoparticles with sizes ranging from 5 to 500 nm, depending on the synthesis conditions. Its uniqueness comprises the non-contact heating of a bulk metal sample under the influence of an electromagnetic field. This method can provide high efficiency at rates up to 200 g/hour of nanoparticles, obtaining a product with the required dispersiveness characteristics. Such parameters guarantee product purity and compliance with the principles of Green Chemistry.

The relevance of the conducted work is the good productivity of high-purity zinc nanoparticles and the ability to control size in a wide range by changing the parameters for the electromagnetic field and the refrigerant gas.

The induction flow levitation method, as described in references [24,25,26], is an aerosol gas phase technique in which nanoparticles form from atoms evaporated from a levitating molten state. This non-contact heating method enables the attainment of higher purity compared to using crucibles [27,28], where the molten metal’s interaction with the crucible material can introduce impurities. Aerosol methods for producing nanoparticles are attractive because they allow control of the process, are cheaper compared to other synthesis methods and allow control of the physical (size, productivity, extent of agglomeration and porosity) and chemical (stoichiometry and surface condition) properties [29].

Thus, aim of this work was to obtain zinc nanoparticles by applying the method of induction flow levitation in an inert gas flow. For this, the work investigated the influence of the nature of refrigerant gas and velocity on the particle size. Methods to study the received particles were SEM, SEM-EDS, TEM, XRF, DLS, porosimetry and ICP-MS.

## 2. Experimental Section

### 2.1. List of Materials

The basic materials were: zinc wire with a diameter of 0.6 mm (99.99%) from Himreaktiv Ltd. (N. Novgorod, Russia); argon and helium(99.9995%) from Monitoring Ltd. (St-Petersburg, Russia).

### 2.2. Experimental Setup and Synthesis Technique

**Figure 1:**
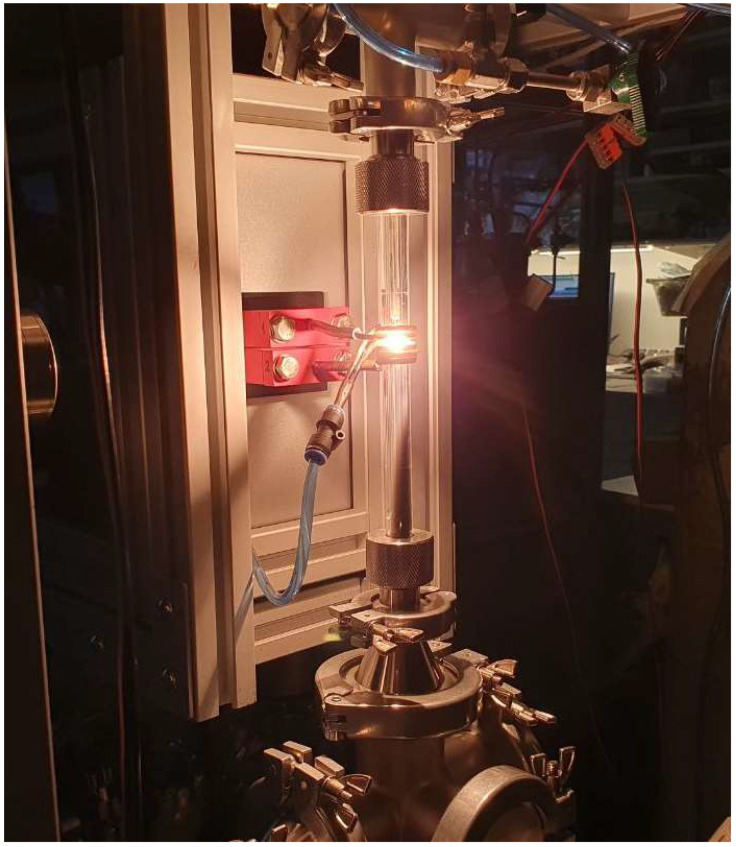
Nanoparticle production in a quartz reactor.

**Figure 2:**
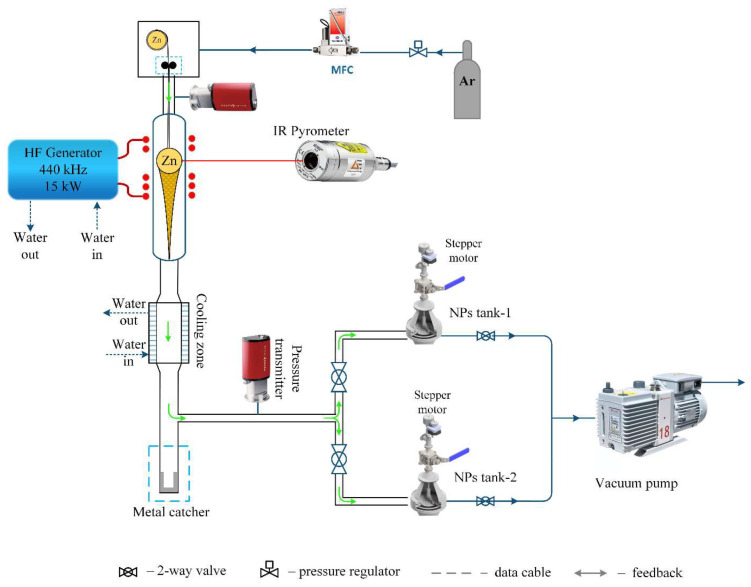
Setup schematic diagram.

The production of zinc nanoparticles was conducted using a setup based on a 10 kW lamp generator operating at a frequency of 440 kHz. The process used 2 g of 8 mm diameter zinc granules with a purity of 99.9%. Inside a quartz reactor, a 0.6 mm diameter zinc wire with a metal ball was suspended. The system underwent a vacuuming process, after which the inert gas argon was introduced. Upon applying voltage to the special configuration induction coil, the sample began melting and started levitating. When the temperature exceeded the melting point, evaporation of the metal atoms occurred with their condensation on an inert gas into nanoparticles with visually monitored flow formation (Figure 1). The nanoparticles were congregated using a fabric filter and subsequently transferred to an inert container for further research. To support a continuous process of nanoparticle production, zinc wire was fed to replace the evaporated metal from the surface of the metal sample. Figure 2 presents the setup diagram.

## 3. Characterization Methods

This work resulted in the production of zinc nanoparticles. The characterization of these nanoparticles involved synthesizing them under specific conditions, including an argon atmosphere at a flow rate of 20 L/min and a pressure of 1000 mbar. The choice of these parameters was determined based on the stability of the melt levitation and the evaporation of the flow of atoms. Economic considerations influencing the resulting nanoparticles’ ultimate cost led to the selection of argon over helium, as these nanoparticles are subsequently spent on catalyst production. The zinc nanoparticles were characterized by the following methods: SEM—scanning electron microscopy; SEM-EDS—energy dispersive spectroscopy; XRD—X-ray diffraction; DLS—dynamic light scattering; TEM—transmission electron microscopy; porosimetry; ICP-MS—inductively coupled plasma mass spectrometry.

SEM, SEM-EDS. The specimens were attached to the sample holder using conductive carbon adhesive tape. Then, to compensate for the electron beam induced charge, they were layered through magnetron sputtering Q150R S (Quorum, East Sussex, UK) with an Au/Pd film (3 nm thick). The visual appearance features and morphology were examined using a scanning electron microscope (Carl Zeiss, Merlin, Jena, Germany) equipped with an in-lens secondary electron detector. The microscope operated at an electron acceleration energy of 5–7 kV, while maintaining a vacuum of approximately 10^−6^ mbar within the chamber. Altami studio 4.0 software was used to process SEM and TEM images to decide the particle size distribution based on at least 100 samples.

Moreover, using a scanning electron microscope JSM-6700F (JEOL, Tokyo, Japan), energy dispersive spectroscopy (EDS) observations were performed, encompassing elements such as Al, C, Mg, Ni, O, Si and Ti, as well as the examination of fracture surfaces of the hybrids, which were prepared under liquid nitrogen. To characterize the elemental distribution of sample, EDS mapping was provided.

TEM, HR-TEM. Transmission electron microscopy (TEM) was conducted using a LIBRA 200 MC Schottky (Carl Zeiss AG, Oberkochen, Germany). To prepare the samples, the nanoparticles dispersed by ultrasonics in dry isopropyl alcohol were deposited onto standard copper grids for a transmission electron microscope. High-resolution TEM images were obtained, and Fourier transformation and filtering techniques were applied to analyze the crystal structure and periodic planes of the nanoparticles. Using ultrasonic dispersion and filtering techniques provided detailed insights into the atomic arrangement within the nanoparticles. The TEM operating conditions, including high voltage (a field emission gun running at 200 kV) and a resolution limit of 0.12 nm, enabled precise and detailed imaging of the nanoparticles, revealing their morphology and structure.

XRD. The XRD-7000 (Shimadzu, Tokyo, Japan) was employed to verify the phase composition of the synthesized nanoparticles. Operating at 40 kV and 30 mA, the measurements were conducted within an angle range of 10–80° (2θ) with a scanning step of 0.02° and an exposure of 0.5 s at each step. A scattering slit RS with a width of 0.3 mm was used at the front part of the detector to capture the XRD data.

DLS. The particle size of the synthesized nanoparticles was analyzed using a NANO-flex device (Microtrac Inc., York, PA, USA) employing the dynamic light backscattering (DLS) method at an 180° angle. This method enables the analysis of particles ranging from 0.3 nm to 10 μm with high resolution. The measurements were conducted in dry isopropyl alcohol and ethylene glycol as a liquid medium for measurement in order to receive stable suspensions with zeta potential values greater than +30 mV STABINO (Microtrac Inc., Montgomeryville, PA, USA). On average, the nanoparticle concentration was 0.05%, the suspension was dispersed in the RAS for 30 min.

SURFACE AREA & POROMETRY. The specific surface area and pore size distribution were determined from absorption/desorption isotherms recorded on an Autosorb IQ instrument (Quantachrome Instruments, Boynton Beach, FL, USA). The samples were prepared and degassed at 100 °C for 6 h in a vacuum of 10^−4^ mbar before commencing the analysis. Interpretation of the adsorption isotherms, in particular the hysteresis loop for different isotherm types, facilitated estimation of the specific surface area using the Brunauer–Emmett–Teller (BET) method and provided an approximate characterization of the pore size distribution, total pore volume, and average specific particle size (ASPS) for spherical objects.

ICP-MS. To study the impurity composition of nanoparticles, an Agilent 7500 CE quadrupole ICP-MS (Agilent Technologies, Omaha, NE, USA) with a Micromist sprayer and a Scott Double Pass spray chamber was spent. Nanoparticles under examination were dissolved in concentrated hydrochloric acid at a rate of 1 μg/L.

## 4. Results and Discussion

Controlling the size of nanoparticles: a special feature of the technology used is the ability to regulate the size of nanoparticles. The refrigerant gas flow rate can affect the nanoparticle size: in this setup at maximum power, it is no more than 30 L/min. The purge gas type also affects nanoparticle growth, depending on its thermal conductivity. For instance, gases with higher thermal conductivity, such as helium (He: 0.152 W/(m K)), can lead to smaller particle sizes due to faster cooling and condensation. In contrast, gases with lower thermal conductivity, like argon (Ar: 0.0164 W/(m K), can result in larger particle sizes.

Another parameter that can be used to control the size of the resulting particles is the applied electromagnetic field power (the power of the EMF generator), on which the temperature of the levitating droplet depends; a decrease in power leads to a temperature decrease, the resulting particle size decreases, but, at the same time, the productivity of the setup decreases. Typically, in the synthesis of nanoparticles, we work at the maximum power of the generator, influencing the nanoparticle size by the refrigerant gas flow rate. Helium is the preferred refrigerant gas, but from an economic point of view we use argon. By reducing the power of the electromagnetic levitation device, the lift force displaces the levitated sample into the lower part of the inductor, where the electromagnetic field (EMF) is denser, resulting in an increased melt temperature. Conversely, by increasing the power, the droplet is mixed into the upper part of the coil, where the EMF density is lower, thereby reducing the sample temperature. This, in turn, alters the evaporation intensity of laminar flow of the metal atoms, thereby influencing the size of nanoparticles and their degree of agglomeration.

Moreover, as the temperature of the levitated droplet increases, the length of the nanoparticle formation process is extended due to a more prolonged cooling period until solid crystal formation. Furthermore, with increased intensity of atom evaporation density, the likelihood of collision among the formed crystallites is increased, thereby forming the final agglomerate of nanoparticles.

Table 1 presents data on the sizes of the resulting Zn nanoparticles at various flow rates and pressure in the system of P = 1000 mbar.

Ar and He were used as refrigerant gases, minimum flow 1 L/min, maximum 30 L/min. Using less than 1 L/min flow rate led to boiling of the levitating droplet and termination of the synthesis. An increase in flow of more than 30 L/min did not allow the performance of the vacuum pump.

The size of the obtained samples in this study was determined from the specific surface area obtained by low-temperature nitrogen porosimetry. The technique for determining the size in this way is described in our work [30].

From Table 1 it can be seen that using helium as a purge gas with higher thermal conductivity led to obtaining smaller zinc nanoparticles. The gas flow rate also affected particle size, with higher flow rates resulting in smaller particles.

**Figure 3:**
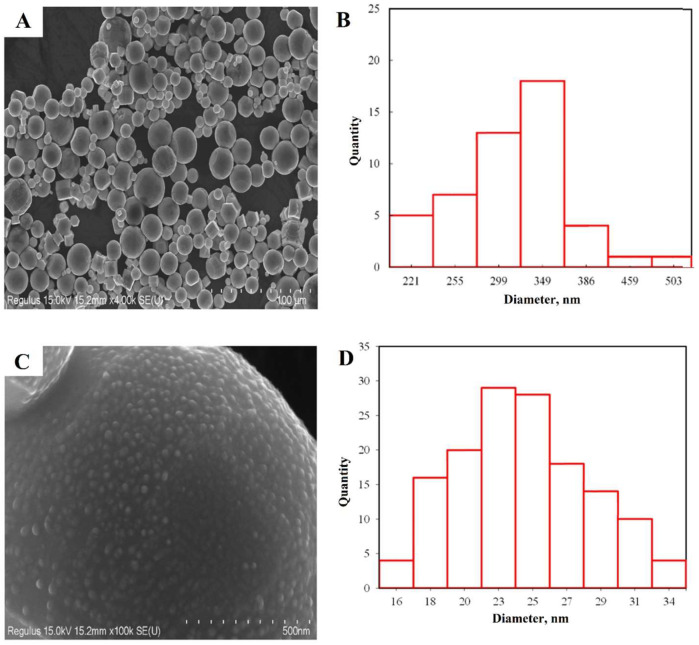
Zinc nanoparticle micrograph at a magnification of 4 k X (**A**), the distribution of particle sizes (**B**), microparticle surface 100 k X (**C**), distribution of grains on the surface of particles (**D**).

SEM and SEM-EDS. Results of Scanning Electron Microscopy (SEM), presented in Figure 3, revealed that the zinc nanoparticles had an average size of 349 nm and exhibited an octahedral shape. This particle size is because of the low melting point (420 °C), the metal droplet is greatly overheated, which leads to a high growth rate of nanoparticles. The zinc sample is microparticles, although nano-sized spherical particles are also present in various areas.

Figure 3B is a micrograph of the surface of one of the zinc nanoparticles. The surface relief has uniform roughness. Statistical analysis (Figure 3D) showed that the average grain size on the surface is 21 nm and suggests that these are primary crystallites. To confirm this assumption, transmission electron microscopy and X-ray diffraction analysis were performed.

**Figure 4:**
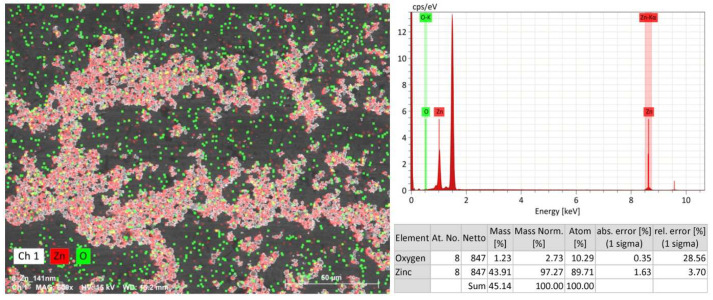
The zinc sample SEM-EDS results (50 µm) by element.

Figure 4 shows the SEM-EDS results for the resulting zinc. At the level of background signals, there were spectra from aluminum (substrate), carbon (conductive tape) and adsorbed oxygen. The background peak values were constant within the error limits, so they were subtracted. Besides the oxygen adsorbed on the surface, an oxide phase could form on the nanoparticle surface, but the obtained values are small and within the error limits. In the zinc sample, the oxygen amount is not above 10 atomic %. Regarding this, it can be assumed that, linked to their high activity, the nanoparticles underwent oxidation during sample preparation.

**Figure 5:**
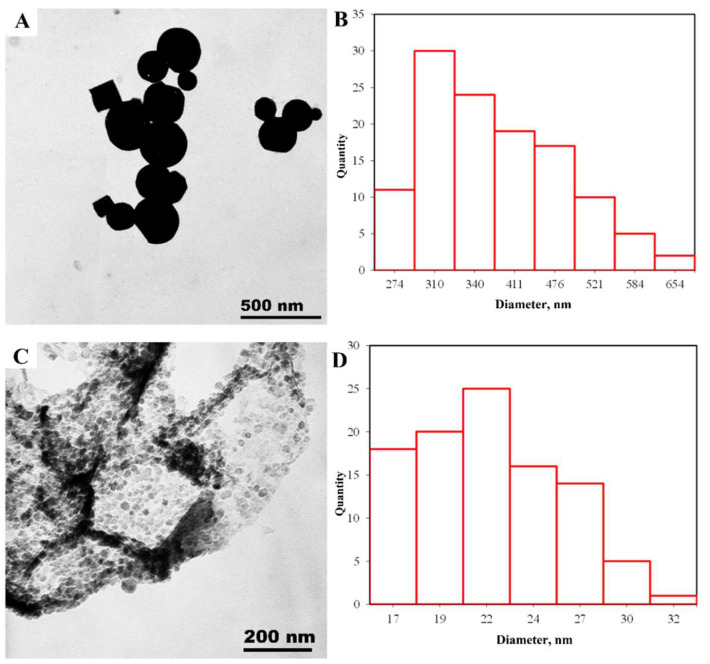
TEM results: (**A**)—micrograph of zinc microparticles; (**B**)—microparticle size distribution; (**C**)—micrograph of nanocrystals forming Zn microparticles; (**D**)—size distribution of nanocrystals.

TEM. The resulting TEM micrographs were subjected to statistical processing to determine the nanoparticle size distribution. In the observed zinc nanoparticle samples (Figure 5) hexagonal structures are formed first, and above 200 nm the particles form a spherical shape. The average particle size is 338 nm.

Figure 5B shows that the nanoparticles are formed from uniform fine-grained zinc nanocrystals with an average size of 21 nm, consistent with the XRD and SEM findings.

**Figure 6:**
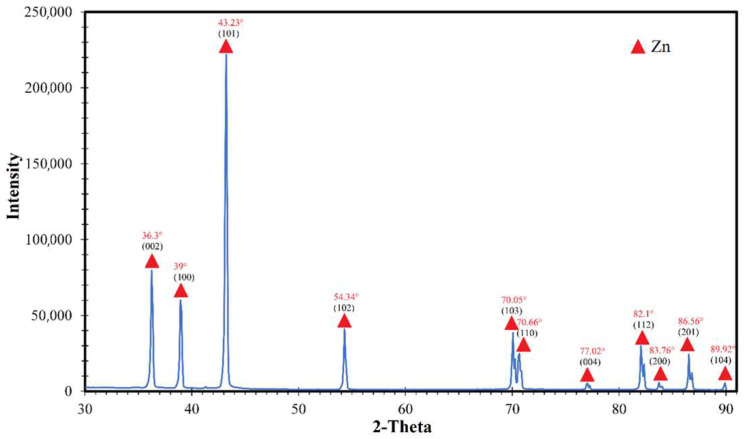
Diffraction pattern of zinc nanoparticles, where ▲—Zn.

XRD. The X-ray diffraction pattern in Figure 6, obtained for a zinc nanoparticles sample, displays peaks indexed by the zinc metal peaks of the crystal structure (structure type P63/mmc). Diffraction peaks at 2θ = 36.3°, 39°, 43.23°, 54.34°, 70.05°, 70.66°, 77.02°, 82.1°, 83.76°, 86.56° and 89.92° correspond to the lattice planes (002), (100), (101), (102), (103), (110), (004), (112), (200), (201) and (104) zinc according to JCPDS No. 96-901-1600. The peak at 21° refers to the polyethylene cuvette. It is important to note that there are no oxide phases, which is somewhat unexpected due to the high tendency of zinc to oxidize.

The average crystallite size was calculated based on the diffraction patterns and the Scherrer equation:

(1) $$d=\frac{K\lambda }{\beta cos\theta }\;,$$

where *d*—average crystallite size; *K*—Scherrer’s constant (*K* = 0.94); *λ*—X-ray radiation wavelength (CuKα radiation wavelength 0.15418 nm); β—width of the reflection at half-height of the peak; *θ*—diffraction angle (Al: 38°, Mg: 34.40°, Ni: 44.45°, Ti: 38°). The average size of primary crystallites, calculated based on the Scherrer equation, using the largest peak, was determined to be 22.1 nm.

DLS. Table 2 presents the measurement results of the received nanoparticles using the DLS method, where N_Peak_ is the peak value of the NP distribution by quantity, N_Mean_ is the average particle size of the NP distribution by quantity, I_Peak_ is the peak value of the NP distribution by intensity, I_Mean_ is the average particle size of the nanoparticle intensity distribution, PDI—polydispersity index. Under the International Organization for Standardization (ISO) for metal nanoparticles, it has been established that nanoparticles with PDI less than 0.5 are considered monodisperse or slightly aggregated nanoparticles with PDI higher than 0.7 or equal to 1 are polydisperse or aggregated. The polydispersity index is a dimensionless parameter, and scaled such that values below 0.05 are uncommon except for highly monodispersion standards. In contrast, values above 0.7 suggest a very wide size distribution, indicating that the sample may not be suitable for dynamic light scattering (DLS) analysis.

**Figure 7:**
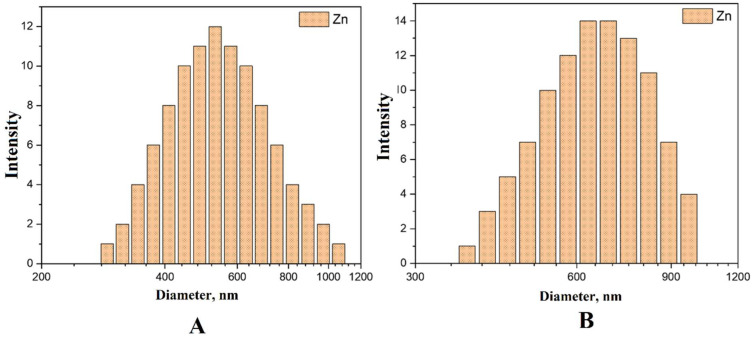
Distribution of Zn NPs obtained by the DLS method: (**A**)—distribution by the number of particles, (**B**)—distribution by particle intensity.

The distribution for zinc nanoparticles in Figure 7 with an average specific size of 538 nm indicates that the NPs have a monodisperse character, but also the presence of a small number of larger particles (about 1 μm).

Porosimetry. The resulting nanoparticle spherical shapes allow the measurement of their average size using the specific surface area (Ssp.), determined through low-temperature nitrogen adsorption. However, due to nanoparticle agglomeration tendencies, the average size obtained from porosimetry is usually larger than that derived from the statistical processing of SEM or TEM images. The porosimetry results are outlined in Table 3.

Table 4 presents the average size obtained by SEM, TEM, XRD, DLS and porosimetry methods to assess convergence. The data indicates that the average specific particle size (ASPS) determined through the specific surface area calculation closely matches the sizes received by other methods. However, the average particle size derived from dynamic light scattering is 2 times larger, indicating that the particles are highly agglomerated.

ICP-MS. The resulting nanoparticle purity was evaluated using inductively coupled plasma mass spectrometry (ICP-MS). Table 5 displays the quantitative values outlining the maximum impurity levels for the elements and also demonstrates purity deviations from the original wire. Thus, the IPL method enables nanoparticle production without introducing other impurities, which can occur in other nanoparticle synthesis approaches. The non-contact nature of sample heating and the single-stage process contribute to achieving high purity, in contrast to methods involving a crucible, which can reduce product purity because of the aggressive nature of molten metal.

The analysis determined the resulting nanoparticles to have a purity of 99.9%, with the quantity of other elements being less than 1000 ppm. Based on the impurity composition analysis results received for the original zinc sample and zinc nanoparticles, it can be concluded that no additional impurities were obtained during the synthesis. Vice versa, the lead, cadmium and nickel concentration has decreased. This is associated with their high segregation coefficient; therefore, they remain in the levitating zinc melt, which means thermal distillation occurs in a certain way.

## 5. Conclusions

This research explored the production of high-purity zinc nanoparticles. The SEM analysis revealed the nanoparticles displayed an octahedral facet, with an average particle size of 349 nm. Such a large particle size is because of the high synthesis temperature, which was approximately 900 °C, very close to the boiling point. Attempts to lower the synthesis temperature by reducing the generator power led to droplet retention cessation by the electromagnetic field, associated with the density and fluidity of zinc. X-ray diffraction analysis confirmed that all peaks belonged to zinc, with a grain size of 21 nm. Porosimetry proved effective for determining nanoparticle size through specific surface area measurements. The resulting nanoparticles exhibited a purity of 99.9%.

## Figures and Tables

**Table 1:** Zinc NP size dependence from different cooling gas flows.

Gas Consumption, L/min	1	5	10	20	30
He	492 nm	402 nm	321 nm	267 nm	141 nm
Ar	634 nm	476 nm	398 nm	350 nm	236 nm

**Table 2:** The metal nanoparticles parameters obtained using the DLS method.

Parameter/NP	Zn
N_Peak_, hm	527.3
N_Mean_, hm	538.2
I_Peak_, hm	660.2
I_Mean_, hm	649.3
PDI	0.049

**Table 3:** Nanoparticle structural characteristics obtained from data for low-temperature nitrogen adsorption at 77 K.

Sample	S_BET_, m^2^/g	V_pore_, sm^3^/g	<D>_pore_, nm	HMA, nm
Zn	11.324	0.039	2.45	94

**Table 4:** Particle sizes obtained from different methods.

Sample	<D>_SEM,_ nm	<D>_TEM,_ nm	<D> _XRD,_ nm	<D>_DLS,_ nm	HMA, nm
Zn	349	338	21	538	94

**Table 5:** Nanoparticles composition received through ICP-MS.

**(Zn_wire_ 0.6 mm 99.9%)**											
Element	ppm										
	Zn, %	Ag	Au	Ba	Ca	K	Na	Pb	Cd	Cu	Ni
ICP-MS	99.9	40	20	15	100	100	40	185	110	400	40
**Zn microparticles**											
Element	Zn, %	ppm									
		Ag	Au	Ba	Ca	K	Na	Pb	Cd	Cu	Ni
ICP-MS	99.9	40	20	15	100	100	40	180	75	400	25

## Data Availability

Data are contained within the article.
